# Diversity and emergence of new variants of African swine fever virus Genotype I circulating in domestic pigs in Nigeria (2016–2018)

**DOI:** 10.1002/vms3.988

**Published:** 2022-11-15

**Authors:** C. Masembe, A. J. Adedeji, A. R. Jambol, R. Weka, V. Muwanika, P. D. Luka

**Affiliations:** ^1^ College of Natural Sciences Makerere University Kampala Uganda; ^2^ Biotechnology Division National Veterinary Research Institute Vom Nigeria; ^3^ College of Agricultural & Environmental Sciences Makerere University Kampala Uganda

**Keywords:** African swine fever, genotype I, multiple variants, Nigeria

## Abstract

**Background:**

African swine fever (ASF) is the most lethal disease of pigs caused by ASF virus (ASFV) with severe economic implications and threat to the swine industry in endemic countries. Between 2016 and 2018, several ASF outbreaks were reported throughout pig producing states in Nigeria.

**Objectives:**

Thereafter, this study was designed to identify the ASFV genotypes responsible for these outbreaks within the study period (2016–2018).

**Methods:**

Twenty‐two ASFV‐positive samples by polymerase chain reaction were selected. The samples were collected during passive surveillance in eight states of Nigeria were characterised using 3 partial genes sequences of the virus namely, *p72* capsid protein of the *B646L*, *p54* envelope protein of *E183L* and the central variable region (CVR) within *B602L* of ASFV.

**Results:**

Phylogenetic and sequences analysis based on *p72* and *p54* revealed ASFV genotype I as the circulating virus. Sequence analysis of the CVR of *B602L* revealed genetic variations with six ASFV tandem repeat sequence (TRS) variants namely, Tet‐15, Tet‐20a, Tet‐21b, Tet‐27, Tet‐31 and Tet‐34, thus increasing the overall genetic diversity of ASFV in Nigeria. Three of the TRS variants, Tet‐21b, Tet‐31 and Tet‐34, were identified for the first time in Nigeria. The new TRS variants of ASFV genotype I were identified in Enugu, Imo, Plateau and Taraba states, while co‐circulation of multiple variants of ASFV genotype I was recorded in Plateau and Benue states.

**Conclusions:**

The high genetic diversity, emergence and increasing recovery of new variants of genotype I in Nigeria should be a concern given that ASFV is a relatively stable DNA virus. The epidemiological implications of these findings require further investigation.

## Graphical abstract

Three partial genes sequences p72 capsid protein of the B646L, p54 envelope protein of E183L, and the central variable region (CVR) within B602L of African swine fever virus from Nigeria were characterised. We found an increase in tandem repeat sequence (TRS) variants, and three variants (Tet‐21b, Tet‐31 and Tet‐34) were identified for the first time in Nigeria.

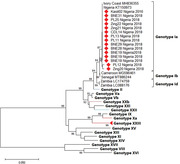

## 1 INTRODUCTION

African swine fever (ASF) is a highly fatal haemorrhagic disease of domestic and wild pigs caused by the ASF virus (ASFV), resulting in severe morbidity and mortality (Odemuyiwa et al., 2000; Penrith & Vosloo, 2009). ASFV is a double‐stranded DNA virus, and the sole member of the genus Asfivirus and family Asfarviridae (Alonso et al., 2018). ASF was first reported in over a hundred years ago in Kenya in 1921, after which the disease was reported in other parts of Africa, and the last few years the disease has spread to Europe, Asia, Oceania and Caribbean countries (Dixon et al., 2020; Montgomery, 1921; Penrith & Kivaria, 2022). The ASFV is transmitted by direct and indirect contact between pigs in the ASFV domestic cycle, soft ticks of the genus: *Ornithodoros* spp. in the tick transmission cycle and via warthogs in the sylvatic cycle (Costard et al., 2013; Jori et al., 2013). Genetic typing of ASFVs is achieved by characterisation of the p72 capsid protein of the B646L gene, full‐length of envelope protein p54 of the E183L gene, and further differentiation of ASFV genotypes can be done using the central variable region (CVR) of the B602L gene (Bastos et al., 2003; Lubisi et al., 2005; Gallardo et al., 2009). Phylogenic studies of B646L gene of ASFV have so far identified 24 genotypes of the virus (Blome et al., 2020). In addition, the serogrouping‐identification approach is also used to distinguish ASFV strains with 8 ASFV serogroups reported based on the EP402R gene (Malogolovkin et al., 2015). Molecular characterisation of B646L, E183L and B602L of the ASFV can be used for investigating the source and extent of outbreaks and possible genetic diversity of circulating viral strains (Lubisi et al., 2005; Malogolovkin et al., 2015). ASFV is a relatively stable DNA virus with low mutation rates and coupled with lack of closely related viruses which reduces the risk of high genetic variation (Dixon et al., 2020; Gaudreault et al., 2020). However, certain regions of the virus such as the CVR are prone to mutations leading to the creation of new ASFV variants (Luka et al., 2016). These new variants might have implications for tracing and tracking the rate of ASF infections across time and space. Pig production activities are carried out in parts in 30 states of the country either as commercial/backyard intensive farms or free‐roaming extensive pig production systems (Fasina et al., 2012). Due to the high demand for live pigs, mobility of these animals is unregulated usually from the north‐central to the Southern coastal parts of the country where farmers and traders can obtain higher prices for their animals (Adedeji et al., 2022). Following the introduction of ASF into Nigeria in 1997, the disease is now endemic in the country with frequent reports of outbreaks in pig producing areas of the country (Odemuyiwa et al., 2000; Owolodun et al., 2010). Previous studies have identified genotype I as the only ASFV circulating in Nigeria and other West African countries (Couacy‐Hymann et al., 2019). But ASFV genotype II was reported in Nigeria recently, which resulted in massive pig mortalities in south western Nigeria (Adedeji et al., 2021). Despite several studies, the epidemiology and probable drivers of the disease in Nigeria are poorly understood (Awosanya et al., 2015; Fasina et al., 2012). Between 2016 and 2018, there was an upsurge in reported cases of ASF in Nigeria affecting eight states of the country. Epidemiological investigations revealed limited understanding of how ASFV spread into and within farming communities. Therefore, this study carried out the genetic characterisation of circulating ASFV to shed light on possible insights on the course and characteristics of these outbreaks.

## 2 MATERIALS AND METHODS

### 2.1 Study area

Nigeria is bordered by Benin Republic, Niger Republic, Chad, Cameroon and the Atlantic Ocean to the southern part. Nigeria's porous international borders with neighbouring countries make trans‐border trading easy with free movement of livestock in both directions. Administratively, Nigeria is divided into 36 states and the Federal Capital Territory (FCT), Abuja.

### 2.2 ASF outbreak investigations and sample collection

**FIGURE 1:**
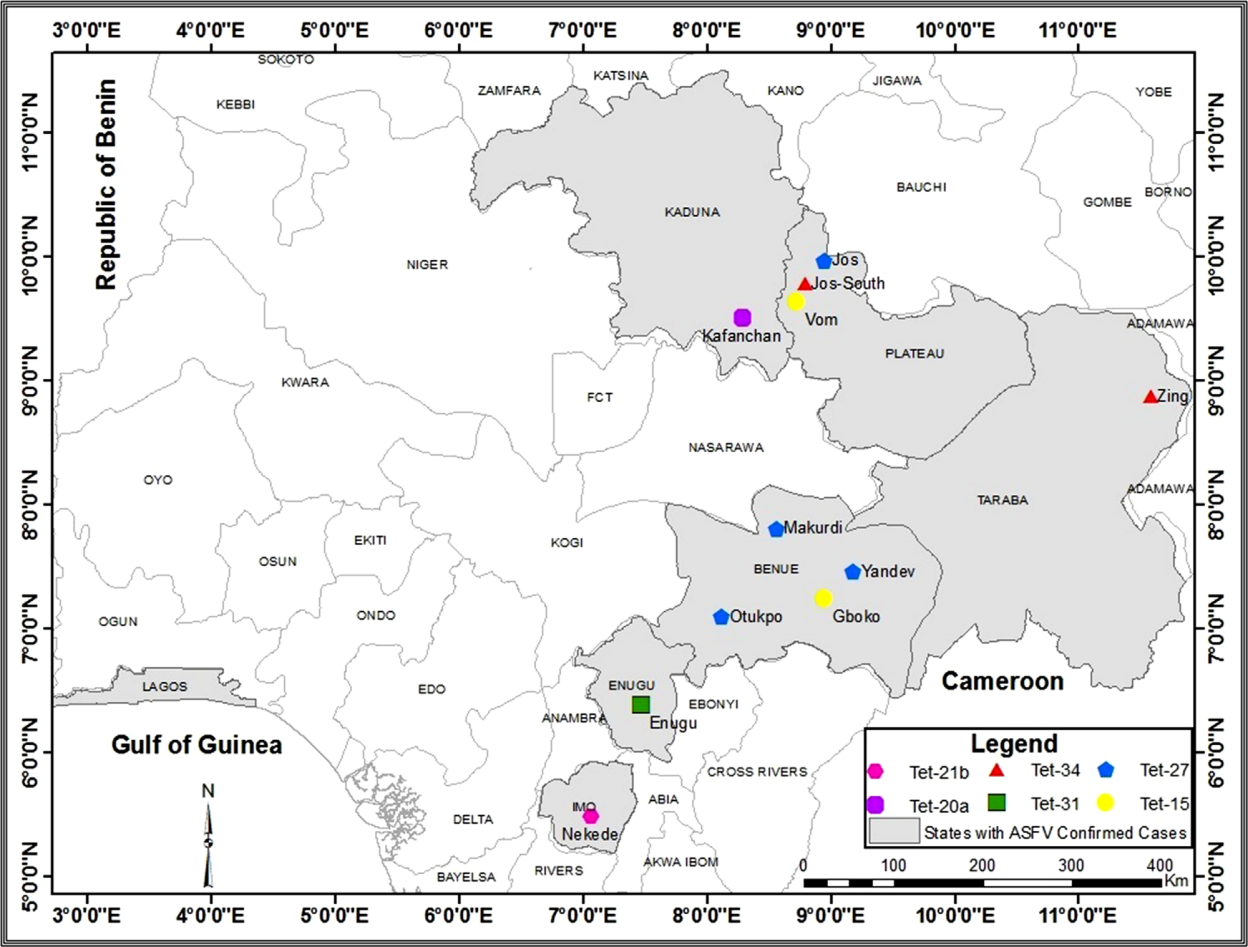
Map of Nigeria showing the distribution of African swine fever virus genotype I variants recovered from outbreaks between 2016 and 2018

Between 2016 and 2018, 37 ASF outbreaks were reported in domestic pigs in 8 states of Nigeria (Figure 1). One hundred ten outbreak samples were collected consisting of whole blood (47) and tissue (63) (liver, spleen and lymph nodes) from 147 pig farms. Samples were collected from intensive/cluster pig farms, and free‐roaming pigs in the affected states of the country. Although, 147 pig farms reported suspected ASF cases, samples were collected in selected farms if they were clusters of pig farms. The affected states were Abia, Benue, Enugu, Kaduna, Imo, Lagos, Plateau and Taraba states (Figure 1, Table 1).

**TABLE 1:** Distribution of African swine fever outbreaks, samples collected, laboratory results in eight states of Nigeria from 2016 to 2018

Year	State	No. of outbreaks	Samples collected	Positive samples by PCR
2016	Benue	1	2	1
	Kaduna	1	2	1
	Plateau	3	10	2
2017	Abia	1	1	1
	Enugu	1	2	2
	Imo	1	2	2
2018	Benue	11	38	11
	Lagos	1	2	2
	Plateau	16	47	14
	Taraba	1	4	2
**Total**		**37**	**110**	**38**

### 2.3 Laboratory analysis

Total genomic DNA was extracted from tissue and blood samples using QIAamp DNA mini kit (Qiagen, Hilden, Germany) following the manufacturer's instructions. ASF diagnosis was carried out by detection of ASFV genome using conventional polymerase chain reaction (PCR) as previously described (Agüero et al., 2003).

### 2.4 Molecular characterisation of African swine fever virus

For ASFV genotyping and assessing the patterns of genetic variation among ASFV positive samples, three regions within the ASFV genome were amplified by PCR and sequenced. These were the C‐terminal end of the *B646L* gene encoding the *p72* protein (Bastos et al., 2003), the full length of *E183L* gene encoding *p54* protein (Gallardo et al., 2009) and CVR within the *B602L* gene as previously described (Lubisi et al., 2005; Phologane et al., 2005; Nix et al., 2006) . The PCR products were purified using MinElute PCR purification kit (Qiagen) as described by the manufacturer's protocol and characterised commercially at Macrogen Inc. (Netherlands Europe) using Sanger sequencing method. The assemblage of sequence reads was carried out using the Staden software package (http://staden.sourceforge.net/) and Bioedit (Hall, 1999) with default settings. Confirmation of sequences type was carried out using the BLAST tool, (https://blast.ncbi.nlm.nih.gov/Blast.cgi). Phylogenetic trees were constructed using MEGA X and inferred using neighbour Joining method for *p72* and Minimum Evolution method for *p54* (Kumar et al., 2018). ASFV genotypes were retrieved from the GenBank for the construction of the phylogenetic trees for both *p72* and *p54*. All ASFV sequences from this study were submitted to the GenBank under accession numbers: OL621859–OL621879 for *B646L* gene, OL621880–OL621896 for *E183L* and OL638971–OL638986 for *B602L*

The sequences of the ASFV *B602L* gene from this study were translated to protein sequences using ExPASy (https://web.expasy.org/translate/) with default settings. The translated amino acid tetramers were matched with published codes as previously reported (Achenbach et al., 2017; Nix et al., 2006). The tandem repeat sequences (TRS) from this study were compared with 25 TRS recovered in Nigeria and other West African countries.

## 3 RESULTS

### 3.1 ASF outbreak investigation

Epidemiological investigations were carried out in eight states. In 2016, ASF was reported in three states of Nigeria including Benue, Kaduna and Plateau states (Table 2). While in 2017, the outbreak occurred three states (Abia, Imo and Enugu) in Eastern Nigeria. The Imo and Abia states ASF outbreak occurred following the introduction of pigs from the Republic of Cameroon into a cluster of pig farms. Imo and Abia states ASF outbreaks resulted in mortality of 73% (2168/2930) and 80% (845/1050), respectively; also the outbreak was enhanced by the activities of butchers who also kept pigs (Table 2). Similarly, ASF outbreaks were reported in 2018 in 3 northern states (Benue, Taraba and Plateau) with common boundaries (Figure 1). In Plateau State, ASF cases were reported between April and November 2018, while in Taraba State, ASF cases were reported in September 2018, affecting 101 pig farmers with a mortality of 45.6% (3354/7360). In addition, widespread ASF outbreaks were reported in four major locations of Gboko, Otukpo, Yandev and Makurdi, all in Benue State from June to December 2018. The affected pig farms were breeder and smallholder intensive farms. Lastly, Lagos State recorded an ASF outbreak close to the Nigeria‐Benin republic international border in an intensive pig farm in 2018 with 75% (40/60) mortality (Table 2).

**TABLE 2:** History of the selected African swine fever positive samples from 2016–2018 outbreaks in Nigeria selected for molecular characterisation

S/No	Sample ID	Type of sample	Year	Location	History
1	KAF‐02	Tissue	2016	Kasit Market, Kaduna State	Sample collected at a pig market
2	PL03	Tissue	2016	Vom, Plateau State	Herd size: 26 pigs, no dead = 3, abortion in sow, fever
3	IMO06	Tissue	2017	Nekede, Imo State	A cluster of seven farms consisting of 60–1000 pigs, a total of 2930 pigs, and 2168 died, and it started after pigs were introduced from Cameroon to one of the farms. Exotic breeds of pigs under intensive management system.
4	ENU09	Tissue	2017	Enugu, Enugu State	Exotic breed of pigs kept under intensive management system
5	PL10	Tissue	2018	Jos Plateau State	Herd size = 22, no dead = 2, cyanosis, anorexia
6	PL11	Tissue	2018	Jos, Plateau State	3‐month‐old piglet, with haemorrhagic diarrhoea,
7	PL12	Tissue	2018	Jos, Plateau State	Herd size: 7, cyanosis and death
8	PL13	Tissue	2018	Jos Plateau State	Sudden deaths of exotic breeds of pigs, cyanosis, fever, anorexia
9	PL14	Tissue	2018	Jos Plateau State	Small‐holder intensive pig, clinical signs include cyanosis, fever, anorexia
11	PL25	Tissue and blood	2018	Vom, Plateau State	Sudden death, herd size: 14; two sick and two died
12	BNE14		2018	Gboko, Benue State	Herd size 55, 4 pigs affected, recumbence, anorexia,
13	BNE28	Blood	2018	Yandev Benue State	An outbreak occurred on intensive pig farm with exotic breeds of pigs, the client unwilling to provide further information.
14	BNE29	Blood	2018	Yandev Benue State	An outbreak occurs on government pig and a client unwilling to provide further information. Pig kept on an intensive production system
15	BNE18	Blood	2018	Makurdi, Benue State	Pigs off feed bloody vomiting and diarrhoea
16	BNE19	Tissue	2018	Oturkpo, Benue State	Herd size = 25; the sudden death of pigs on an institutional farm
17	ZNG 20	Tissue	2018	Zing, Taraba State	The outbreak affected 101 herds of pigs; 3354 pigs died. This occurs in a rural community where pigs were kept under semi‐intensive or extensive production system. Average herd size 2–150 pigs. ASF mortality rate was 45.6% (3354/7360)
17	ZNG21	Tissue	2018	Zing, Taraba State	Outbreak affected 101 herds of pigs; 3354 pigs died. This occurred in a rural community where pigs were kept under semi‐intensive or extensive production system. Herd size 2–150 pigs. ASF mortality rate was 45.6% (3354/7360)
18	ZNG23	Tissue	2018	Zing, Taraba State	Outbreak affected 101 herds of pigs; 3354 pigs die. This occurred in a rural community where pigs were kept under semi‐intensive or extensive production system. Average herd size 2–150 pigs. ASF mortality rate was 45.6% (3354/7360)
19	BNE31	Blood	2018	Yandev, Benue	Outbreak occurs on a government pig farm, and the client unwilling to provide further information. Pig kept on an intensive production system
20	BEN02	Blood	2018	Yandev, Benue State	Outbreak occurs on a government pig farm, and the client unwilling to provide further information. Pig kept on an intensive production system
21	LA1	Tissue	2018	Lagos, Nigeria‐Benin republic border	Anorexia, cyanosis, still‐birth, vomiting, herd size: 60, deaths: 40

### 3.2 Laboratory results

From the 110 samples collected within the 3 years study period, laboratory analysis by conventional PCR indicated that in 42.5% (38/110) of samples collected ASFV was detected. The positive samples were distributed in the respective states as follows: Abia (1) Benue (12), Enugu (2), Kaduna (1), Imo (2), Lagos (2), Plateau (16) and Taraba (2).

### 3.3 Phylogenetic analysis of *B646L* of ASFV

**FIGURE 2:**
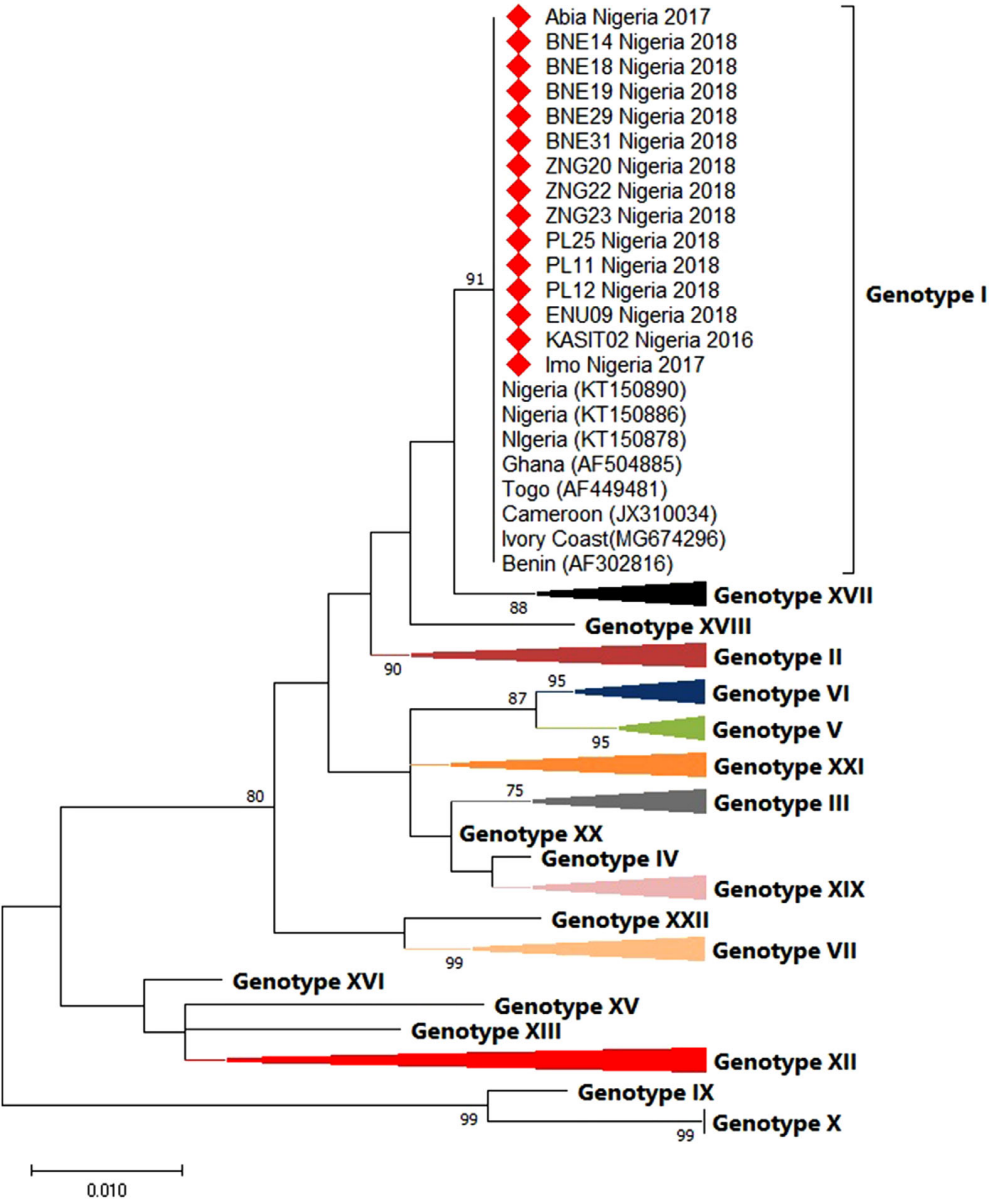
Phylogenetic tree of partial *p72* protein encoded in *B646L* gene of Nigerian ASFV sequences (2016–2018). The tree was constructed in MEGA X using the neighbour joining at 1000 bootstrap replicates. The Nigerian ASFV isolates from this study are marked red.

The *p72* of *B646L* gene sequences generated from this study when compared with other sequences in the GenBank revealed a 99%–100% similarity to ASF viruses from Cameroon (MG596409, MG596420) and Ivory Coast (MG674296) using the BLAST search tool. Phylogenetic analysis of the Nigerian sequences with representatives of the 18 genotypes showed sequences generated in this study clustered with genotype 1 (Figure 2, Table 3).

**TABLE 3:** African swine fever viruses p72 and p54 sequences used for phylogenetic analysis

Country (Genbank Accession number)	p72	References	Country (Genbank Accession number)	p54	References
Benin AF302816	I	Bastos et al. (2003)	Ivory Coast (MH836355)	Ia	Couacy‐Hymann et al. (2018)
Ghana (KT368177)	I	Bastos et al. (2003)	Cameroon (MG596461)	Ia	Wade et al. (2019)
Ivory Coast (MG674296)	I	Couacy‐Hymann et al. (2018)	Nigeria (KT150973)	Ia	Luka et al. (2016)
Cameroon (MG596420)	I	Wade et al. (2019)	Senegal (MT886244)	Ic	Minoungou et al. (2021)
Togo (AF449481)	I	Bastos et al. (2003)	DRC (FJ174423)	Ib	Gallardo et al. (2009)
Madagascar (AF270706)	II	Bastos et al. (2004)	Zambia LC174759	Id	Simulundu et al. (2017)
Mozambique(AY351518)	II	Bastos et al. (2004)	Zambia LC088176	Id	Simulundu et al. (2017)
Botswana (AF504886)	III	Bastos et al. (2003)	Madagascar(KC662387)	II	Couacy‐Hymann et al. (2018)
South Africa (AF449477)	IV	Bastos et al. (2003)	Mozabique (EU874371)	Va	Couacy‐Hymann et al. (2018)
Malawi (AY494553)	V	Lubisi et al. (2005	Mozambique (FJ174422)	Vb	Couacy‐Hymann et al. (2018)
Mozambique (AF270709)	V	Bastos et al. (2004)	Zambia (KF736412)	VIII	Couacy‐Hymann et al. (2018)
Mozambique (AF270710)	VI	Bastos et al. (2003)	Uganda (FJ174430)	Xa	Gallardo et al. (2009)
Mozambique (AF270711)	VI	Bastos et al. (2003)	Zanmbia (EU874331)	XI	Couacy‐Hymann et al. (2018)
South Africa (AF302818)	VII	Bastos et al. (2003)	Zambia (EU874357)	XIII	Couacy‐Hymann et al. (2018)
South Africa (DQ250121)	VII	Boshoff et al. (2007)	Zambia (EU874330)	XIV	Couacy‐Hymann et al. (2018)
DRC (MN609932)	IX	Patrick et al. (2020)	Tanzania (GQ410768)	XV	Misinzo et al. (2011)
Kenya (HM745257)	X	Gallardo et al. (2011)	Zambia (KF015947)	XVI	Couacy‐Hymann et al. 92018)
Malawi (AY351561)	XII	Lubisi et al. (2005)	Zambia (KF015915)	XVII	Couacy‐Hymann et al. (2018)
Zambia (AY351543)	XII	Lubisi et al. (2005)	South Africa (EU874381)	XXII	Couacy‐Hymann et al. (2018)
Zambia (AY351542)	XIII	Lubisi et al. (2005)	Ethiopia (KT795370)	XXIII	Achenbach et al. (2017)
Zambia (AY351555)	XV	Lubisi et al. (2005)	Ethiopia (KT795369)	XXIII	Achenbach et al. (2017)
Tanzania(AY494550)	XVI	Lubisi et al. (2005)			
Zimbabwe (DQ250119)	XVII	Boshoff et al. (2007)			
Zimbabwe (KC662376)	XVII	Couacy‐Hymann et al. (2018)			
Nambia (DQ250122)	XVIII	Boshoff et al. (2007)			
South Africa (AF302812)	XIX	Boshoff et al. (2007)			
South Africa (DQ250112)	XIX	Boshoff et al. (2007)			
South Africa (DQ250118)	XIX	Boshoff et al. (2007)			
South Africa AY261363	XX	Couacy‐Hymann et al. (2018)			
South Africa (DQ250125)	XXI	Boshoff et al. (2007)			
South Africa (DQ250117)	XXII	Boshoff et al. (2007)			

### 3.4 Phylogenetic analysis of *E183L* (p54) of ASFV

**FIGURE 3:**
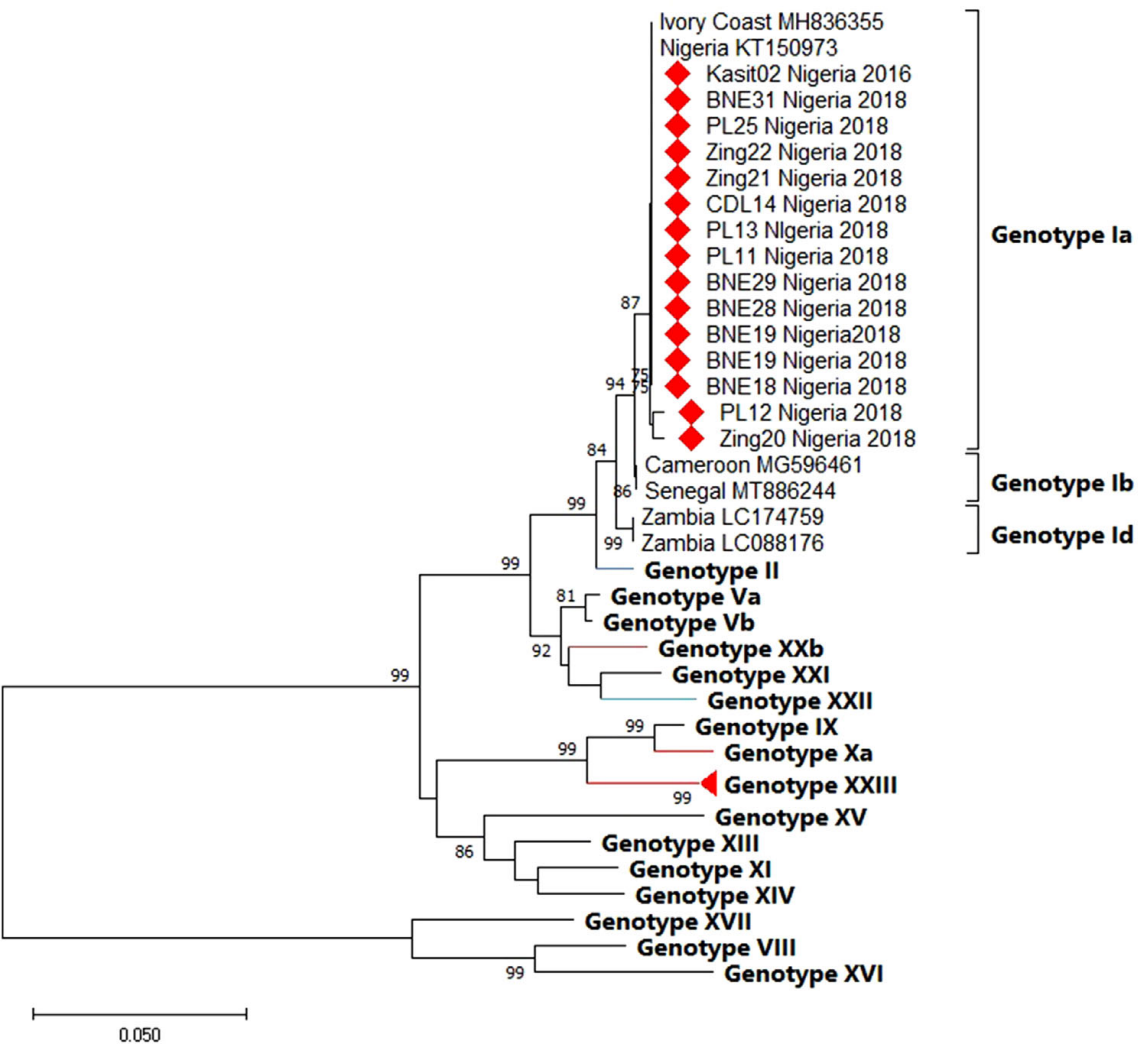
Phylogenetic tree of full‐length *E183L* gene encoding the *p54* protein of Nigeria ASFV isolates from 2016 to 2018. The tree was constructed using MEGA X inferred by Minimum Evolution Method at 1000 bootstrap replicates. The Nigerian ASFV isolates of this study are noted in diamond and cluster with Genotype Ia.

The phylogenetic analysis of the *p54* protein of ASFV revealed that all Nigerian sequences clustered with some sequences from West African countries belonging to genotype 1a (Figure 3). The phylogenetic tree was constructed using sequences of 16 genotypes retrieved from the Genbank (Table 3). Comparison of sequences from this study and recent sequences from other West African countries showed 99%–100% similarity with sequences from Cameroon (MG596464, MG596468), Ivory Coast (MH836355) and Mali (MT886241).

### 3.5 Sequence analysis of the CVR of *B602L*

Sequence analysis of the CVR of the *B602L* gene recovered six ASFV TRS variants named as: Tet‐15, Tet‐20a, Tet‐21, Tet‐27b, Tet‐31 and Tet‐34 (Table 4). ASF variants Tet‐15, Tet‐20a and Tet‐27 had been previously reported in Nigeria (Table 4). While Tet‐21b, Tet‐31 and Tet‐34 were the new variants that are being reported for the first time in Nigeria and West Africa in this study. The Nigerian Isolates KASIT_2, recovered from a live pig market in Katsit, Kaduna State is similar to Cameroonian ASFV (AF513045) (Table 4). Plateau State had the highest number (3) of co‐circulating ASFV TRS variants (Tet‐15, Tet‐27 and Tet‐34) followed by Benue State with two (Tet‐15 and Tet‐27). Interestingly, Tet‐34 that was circulating in pig farms between April and November 2018 in Plateau State was also recovered in Zing, Taraba State in September 2018 possibly introduced from Plateau State via movement of live pigs. ASFV TRS variant Tet‐31 recovered from Enugu State has not been previously reported in Nigeria, but the source of infection is unknown due to limited epidemiological information. Tet‐15 was the only ASFV genotype I variant recovered in two separate years of this study, it was circulating in two states (Plateau and Benue). Tet‐21b recovered from pigs in Imo State which were reported to have been introduced from Cameroon but has not reported in Cameroon.

**TABLE 4:** CVR profiles of *B602L* gene of African swine fever virus from outbreaks between 2016 and 2018 in Nigeria

	Sample ID	Location/state	Year	Accession number	Tetramer	Reference	CVR profile
1	KASIT_ 02	Kafanchan, Kaduna State	2016	OL638984	Tet‐20a	This study	ABNAAAACBNAAAACBNAFA
2	PL03	K‐Vom, Plateau State	2016	OL638986	Tet‐15	This study	ABNABNAAAAACBNA
3	IMO06	Nekede, Imo State	2017	OL638985	*Tet‐21b	This study	ABNAAAAACBNAAAACBNAFA
4	ENU09	Enugu, Enugu	2017	OL638983	*Tet‐31	This study	ABNAAAACBNAAAAAACBNAAAAAACBNAFA
5	BNE14	Gboko Benue State	2018	OL638971	Tet‐15	This study	ABNABNAAAAACBNA
6	PL10	Jos, Plateau State	2018	OL638980	Tet‐15	This study	ABNABNAAAAACBNA
7	BEN19	Otukopo, Benue State	2018	OL638972	Tet‐27	This study	ABNAAAACBNAAAACBNAAAACBNAFA
8	PL 20	Jos Plateau State	2018	OL638976	Tet‐27	This Study	ABNAAAACBNAAAACBNAAAACBNAFA
9	ZNG21	Zing, Taraba State	2018	OL638977	*Tet‐34	This study	ABNAAAACBNAAAAAAAAACBNAAAAAACBNAFA
10	PL24	Jos, Plateau State	2018	OL638978	*Tet‐34	This study	ABNAAAACBNAAAAAAAAACBNAAAAAACBNAFA
11	PL12	Jos, Plateau State	2018	OL638982	*Tet‐34	This study	ABNAAAACBNAAAAAAAAACBNAAAAAACBNAFA
12	PL13	Jos, Plateau State	2018	OL638975	*Tet‐34	This study	ABNAAAACBNAAAAAAAAACBNAAAAAACBNAFA
13	PL14	Jos, Plateau State	2018	OL638981	*Tet‐34	This study	ABNAAAACBNAAAAAAAAACBNAAAAAACBNAFA
14	PL25	Jos, Plateau State	2018	OL638979	*Tet‐34	This study	ABNAAAACBNAAAAAAAAACBNAAAAAACBNAFA
15	BNE28	Yandev, Benue State	2018	OL638974	Tet‐27	This study	ABNAAAACBNAAAACBNAAAACBNAFA
16	BNE18	Yandev, Benue State	2018	OL638973	Tet‐27	This study	ABNAAAACBNAAAACBNAAAACBNAFA

* The newly recovered ASF TRS variants from this study.

*Notes*. CVR codes as previously described: CAST/CVST/CTST = A, CADT/CTDT = B, GAST/GANT = C, CASM = D, CANT = F, CTNT = G, NEDT = M, NVDT/NVGT/NVNT = N, NANI/NADI/NASI = O, RAST = H, SAST = S, NVNT = T, NAST/NADT/NANT = V and SADT/SVDT = W.

## 4 DISCUSSION

This study presents an update on molecular data of ASFV responsible for outbreaks in Nigeria between 2016 and 2018. Results of phylogeny of ASFV sequences obtained in this study identified genotype I Similar previous studies in the country (Owolodun et al., 2010; Luka et al., 2016). However, sequence analysis of the hypervariable CVR encoded within *B602L* gene revealed six ASFV genotype I variants, three of which were new (Tet‐21b, Tet‐31 and Tet‐34) and never reported in Nigeria. Although, Tet‐31 has been recently reported in Burkina Faso (Minoungou et al., 2021). The TRS Tet‐31 newly identified in Nigeria in this study was recovered in pigs reported to be imported from Cameroon; however, it is yet to be reported in that country. Earlier genetic studies had recovered 18 ASFV genotype I variants in Nigeria (Adedeji et al., 2022; Owolodun et al., 2010; Luka et al., 2016) (Table 4). With 3 newly recovered variants (Tet‐21b, Tet‐31 and Tet‐34) in this study, 21 ASFV genotype I TRS variants have now been recovered in Nigeria. In this study, Tet‐15, Tet‐27 and Tet‐34 were the most widely distributed ASFV variants co‐circulating in Benue and Plateau states. (Table 4, Figure 1). Previously, Tet‐36 was the most common variant between 2003 and 2007 and Tet‐20b was a common variant from 2007 to 2015 (Luka et al., 2016). However, Tet‐15 was first reported in Kaduna State in 2014 at a live pig market, but the variant has now spread to other states as shown by the results from this study (Table 4). It is likely Tet‐15 spread from the live pig market in Kaduna State to Benue and Plateau states via trading of live pigs. Likewise, Tet‐27 was first recovered in 2003 and was recovered in several sites in Benue State in 2018 (Luka et al., 2016; Owolodun et al., 2010) (Table 4). Multiple variants of ASFV genotype I have been recovered in some countries in West and Central Africa. For instance, multiple ASFV genotype I variants have been recovered in Ivory Coast (4), Benin (4), Burkina Faso (10), Ghana (6) and Cameroon (4) (Luka et al., 2016; Couacy‐Hymann et al., 2019; Wade et al., 2019; Minoungou et al., 2021; Sidi et al., 2022). These findings suggest the CVR of *B602L* of ASFV genotype I is prone to mutations in West and Central Africa. Furthermore, these mutations occur despite no documented evidence of the presence of sylvatic or tick ASFV transmission cycle in West and Central Africa (Luka et al., 2017). Interestingly, 7 ASFV genotype 1 TRS variants have been detected in two or more West and Central African countries, which further shed light on the possible spread of ASFV in the region. For instance, 3 genotype I variants (Tet‐22, Tet‐29 and Tet‐36) recovered in Nigeria have also been reported in the Benin Republic (Adedeji et al., 2022; Couacy‐Hymann et al., 2019). Likewise, Tet‐20 and Tet‐21 have been reported both in Nigeria and Cameroon (Owoludun et al., 2003; Wade et al., 2019). Hitherto, reports have shown that the introduction and rapid spread of ASFV were due to live pigs trading and free movement of pigs across international borders in the West African sub‐region (Brown et al., 2017; El‐Hicheri 1998). ASF outbreaks frequently occur in Nigeria with severe clinical outcomes, thereby affecting pig farmers' financial income and threatening food security. In this study, ASF outbreaks were reported in eight out of 30 pig‐producing states of Nigeria (Figure 1). Although the number of outbreaks may be higher but were underreported due to a lack of financial compensation to pig farmers. Rather, farmers rapidly sell‐off or slaughter sick pigs leading to further spread of the disease in Nigeria (Fasina et al., 2010). Epidemiological data collected in this study showed that basic biosecurity measures such as proper quarantine before the introduction of new stock and traffic control were not observed, leading to the introduction and spread of ASF in the affected pig farms. Several risk factors have been identified as being responsible for the spread of ASFV in Nigeria, namely, poor husbandry system, live pigs trading and slaughtering of pigs on the farm and movement of ASF infected and recovered animals (Fasina et al., 2012). Other factors include external sourcing of replacement stock, presence of ASF‐infected farms within the neighbourhood of other farms and exchange of feed and farm tools by farmers and their workers (Olugasa & Ijagbone, 2007; Awosanya et al., 2015). This study further confirms the importance of these risk factors in the spread and sustenance of the virus in the pig populations in Nigeria.

## 5 CONCLUSION

This study elucidates the continuous spread and emergence ASFV genotype I TRS variants in pig farming areas in Nigeria within 3 years of the study (2016–2018). Although phylogeny revealed genotype I, however, six variants were recovered following sequence analysis of CVR of *B602L* gene. This study presents the first report of three of the variants that are co‐circulating in four states in Nigeria. The increasing recovery of new variants of genotype I in Nigeria should be a source of serious concern, particularly for a stable DNA virus like ASFV. But the epidemiological implications of the findings are unknown and need further investigation.

## AUTHOR CONTRIBUTIONS

Conceptualisation, data curation, formal analysis, investigation, methodology, administration, supervision, writing original draft, writing, review and editing (Masembe Charles; Adedeji Adeyinka Jeremy; Luka Dachung Pam; Muwanika Vincent). Investigation, visualisation and writing (Jambol Anvou Rachael and Weka Rebecca).

## CONFLICT OF INTEREST

The authors of this study declare that they have no conflict of interests.

### ETHICAL APPROVAL

This study was approved by National Veterinary Research Institute Animal Ethics Committee Vom, Nigeria (AEC/03/26/16).

### PEER REVIEW

The peer review history for this article is available at https://publons.com/publon/10.1002/vms3.988

## Data Availability

The data that support the findings of this study are openly available in GenBank at https://www.ncbi.nlm.nih.gov.
